# *N*,*N*-Dicyclo­hexyl­cyclo­hexa­ne­carboxamide

**DOI:** 10.1107/S1600536812027766

**Published:** 2012-06-27

**Authors:** Sohail Saeed, Naghmana Rashid, Rizwan Hussain, Jerry P. Jasinski, Amanda C. Keeley

**Affiliations:** a Chemistry Department, Research Complex, Allama Iqbal Open University, Islamabad 44000, Pakistan; b National Engineering & Scientific Commission, PO Box 2801, Islamabad, Pakistan; c Department of Chemistry, Keene State College, 220 Main Street, Keene, NH 03435-2001, USA

## Abstract

In the title compound, C_19_H_33_NO, all three cyclo­hexane rings adopt chair conformations. The crystal packing features weak C—H⋯O inter­actions, forming a supra­molecular chain along the *c* axis.

## Related literature

For related studies of *N*-substituted benzamides, see: Saeed *et al.* (2011*a* ▶,*b* ▶). For a related structure, see: Saeed *et al.* (2012 ▶). For puckering parameters, see: Cremer & Pople (1975 ▶).
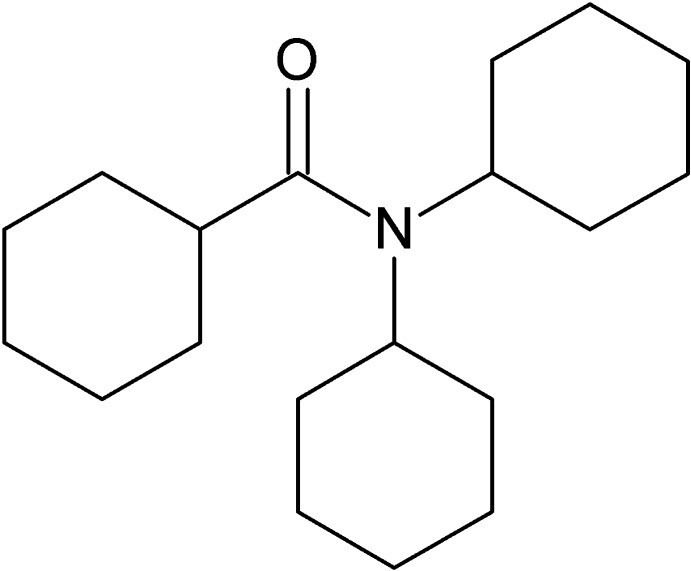


## Experimental

### 

#### Crystal data

C_19_H_33_NO
*M* *_r_* = 291.46
Monoclinic, 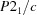

*a* = 9.8237 (3) Å
*b* = 16.8736 (5) Å
*c* = 10.8886 (3) Å
β = 102.890 (3)°
*V* = 1759.42 (10) Å^3^
*Z* = 4
Cu *K*α radiation
μ = 0.50 mm^−1^
*T* = 173 K
0.44 × 0.38 × 0.18 mm

#### Data collection

Agilent Xcalibur Eos Gemini diffractometer
Absorption correction: multi-scan (*CrysAlis PRO*; Agilent, 2012 ▶) *T*_min_ = 0.940, *T*_max_ = 1.000
10594 measured reflections
3369 independent reflections
3023 reflections with *I* > 2σ(*I*)
*R*_int_ = 0.028

#### Refinement

*R*[*F* ^2^ > 2σ(*F* ^2^)] = 0.044
*wR*(*F* ^2^) = 0.123
*S* = 1.05
3369 reflections
190 parameters
H-atom parameters constrained
Δρ_max_ = 0.25 e Å^−3^
Δρ_min_ = −0.19 e Å^−3^

### 

Data collection: *CrysAlis PRO* (Agilent, 2012 ▶); cell refinement: *CrysAlis PRO*; data reduction: *CrysAlis RED* (Agilent, 2012 ▶); program(s) used to solve structure: *SHELXS97* (Sheldrick, 2008 ▶); program(s) used to refine structure: *SHELXL97* (Sheldrick, 2008 ▶); molecular graphics: *SHELXTL* (Sheldrick, 2008 ▶); software used to prepare material for publication: *SHELXTL*.

## Supplementary Material

Crystal structure: contains datablock(s) I, global. DOI: 10.1107/S1600536812027766/tk5114sup1.cif

Structure factors: contains datablock(s) I. DOI: 10.1107/S1600536812027766/tk5114Isup2.hkl

Supplementary material file. DOI: 10.1107/S1600536812027766/tk5114Isup3.cml

Additional supplementary materials:  crystallographic information; 3D view; checkCIF report

## Figures and Tables

**Table 1:** Hydrogen-bond geometry (Å, °)

*D*—H⋯*A*	*D*—H	H⋯*A*	*D*⋯*A*	*D*—H⋯*A*
C2—H2⋯O1^i^	0.98	2.44	3.3861 (13)	163

Symmetry code: (i) 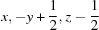.

**Fig. 1.**
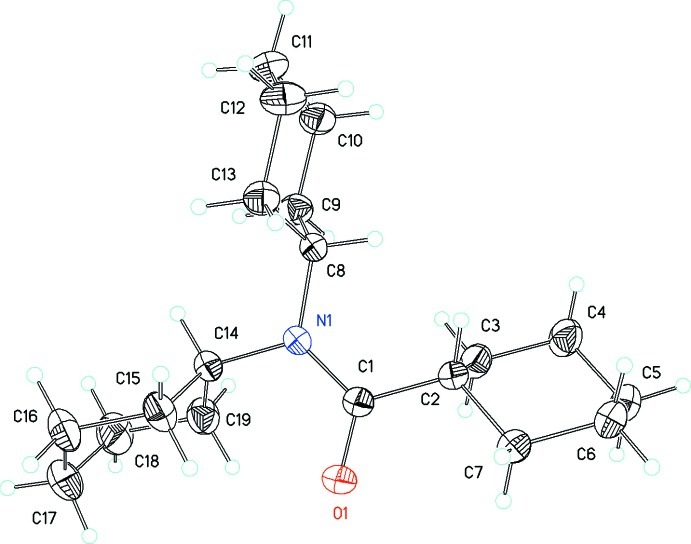
Molecular structure of the title compound showing the atom labeling scheme and 50% probability displacement ellipsoids.

**Fig. 2.**
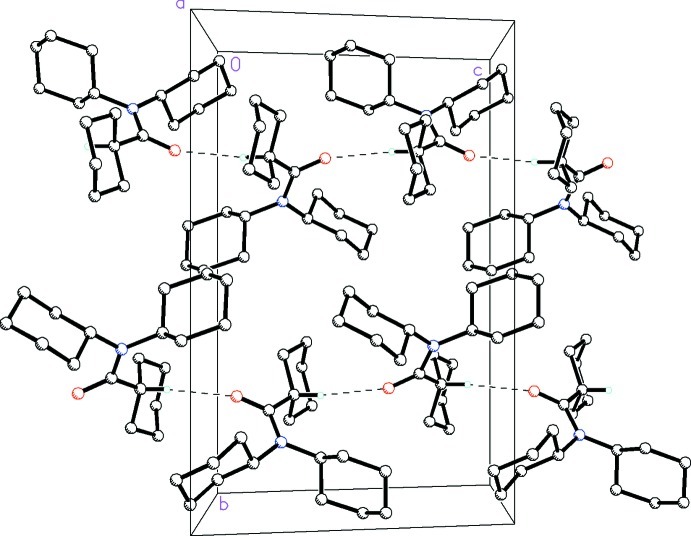
Packing diagram of the title compound viewed along the a axis. Dashed lines indicate weak C—H···O interactions forming a 1-D chain along the c axis. Remaining H atoms have been removed for clarity.

## supplementary crystallographic information

### Comment

In connection with on-going studies into *N*-substituted benzamides (Saeed *et al.*, 2011*a*; Saeed *et al.*, 2011*b*), we recently determined the crystal structure of *N*-(4-bromophenyl)-3,5-dinitrobenzamide (Saeed *et al.*, 2012). In this paper we present the crystal structure of the title compound, (I).

In (I), Fig. 1, all three cyclohexane groups adopt a chair conformation with puckering parameters Q, θ and φ of 0.5850 (14) Å, 0.00 (14)°, and 320 (10)° (C2–C7); 0.517 (13) Å, 178.40 (13)° and 237 (4)° (C8–C13); 0.5747 (15) Å, 0.54 (15)°, and 120 (14)° (C14–C19), respectively (Cremer & Pople, 1975). Crystal packing is stabilized by weak C—H···O intermolecular interactions (Table 1) forming a 1-D supramolecular chain along the *c* axis (Fig. 2).

### Experimental

To a 250 ml round bottom flask fitted with a condenser was added dicyclohexyl amine (0.01 mol), dichloromethane (15 ml) and triethylamine (0.5 ml) with magnetic stirring. Cyclohexanoyl chloride (0.01 mol) was added gradually. The reaction mixture was stirred at room temperature for 1 h and then refluxed for 2 h. The product precipitated as white powder, which was washed three times with water. Recrystallization from ethyl acetate produced the crystals of the title compound.

### Refinement

All of the H atoms were placed in their calculated positions and then refined using the riding model with C—H lengths of 0.98 Å (CH) or 0.97 Å (CH_2_). The isotropic displacement parameters for these atoms were set to 1.20–1.21 (CH) or 1.18–1.20 (CH_2_) times *U*_eq_ of the parent atom.

### Crystal data

**Table:**

C_19_H_33_NO	*F*(000) = 648
*M**_r_* = 291.46	*D*_x_ = 1.100 Mg m^−^^3^
Monoclinic, *P*2_1_/*c*	Cu *K*α radiation, λ = 1.54184 Å
Hall symbol: -P 2ybc	Cell parameters from 5299 reflections
*a* = 9.8237 (3) Å	θ = 4.2–71.1°
*b* = 16.8736 (5) Å	µ = 0.50 mm^−^^1^
*c* = 10.8886 (3) Å	*T* = 173 K
β = 102.890 (3)°	Chunk, colourless
*V* = 1759.42 (10) Å^3^	0.44 × 0.38 × 0.18 mm
*Z* = 4	

### Data collection

**Table:**

Agilent Xcalibur Eos Gemini diffractometer	3369 independent reflections
Radiation source: Enhance (Cu) X-ray Source	3023 reflections with *I* > 2σ(*I*)
Graphite monochromator	*R*_int_ = 0.028
Detector resolution: 16.1500 pixels mm^-1^	θ_max_ = 71.3°, θ_min_ = 4.6°
ω scans	*h* = −11→9
Absorption correction: multi-scan (*CrysAlis PRO*; Agilent, 2012)	*k* = −20→18
*T*_min_ = 0.940, *T*_max_ = 1.000	*l* = −13→13
10594 measured reflections	

### Refinement

**Table:**

Refinement on *F*^2^	Primary atom site location: structure-invariant direct methods
Least-squares matrix: full	Secondary atom site location: difference Fourier map
*R*[*F*^2^ > 2σ(*F*^2^)] = 0.044	Hydrogen site location: inferred from neighbouring sites
*wR*(*F*^2^) = 0.123	H-atom parameters constrained
*S* = 1.05	*w* = 1/[σ^2^(*F*_o_^2^) + (0.073*P*)^2^ + 0.3063*P*] where *P* = (*F*_o_^2^ + 2*F*_c_^2^)/3
3369 reflections	(Δ/σ)_max_ < 0.001
190 parameters	Δρ_max_ = 0.25 e Å^−^^3^
0 restraints	Δρ_min_ = −0.19 e Å^−^^3^

### Special details

**Table:**

Experimental. Agilent Technologies, (2012). CrysAlisPro, Version 1.171.35.21 (release 20-01-2012 CrysAlis171 .NET) (compiled Jan 23 2012,18:06:46) Empirical absorption correction using spherical harmonics, implemented in SCALE3 ABSPACK scaling algorithm.
Geometry. All e.s.d.'s (except the e.s.d. in the dihedral angle between two l.s. planes) are estimated using the full covariance matrix. The cell e.s.d.'s are taken into account individually in the estimation of e.s.d.'s in distances, angles and torsion angles; correlations between e.s.d.'s in cell parameters are only used when they are defined by crystal symmetry. An approximate (isotropic) treatment of cell e.s.d.'s is used for estimating e.s.d.'s involving l.s. planes.
Refinement. Refinement of *F*^2^ against ALL reflections. The weighted *R*-factor *wR* and goodness of fit *S* are based on *F*^2^, conventional *R*-factors *R* are based on *F*, with *F* set to zero for negative *F*^2^. The threshold expression of *F*^2^ > σ(*F*^2^) is used only for calculating *R*-factors(gt) *etc*. and is not relevant to the choice of reflections for refinement. *R*-factors based on *F*^2^ are statistically about twice as large as those based on *F*, and *R*- factors based on ALL data will be even larger.

### Fractional atomic coordinates and isotropic or equivalent isotropic displacement parameters (Å^2^)

**Table:**

	*x*	*y*	*z*	*U*_iso_*/*U*_eq_	
N1	0.32349 (9)	0.15353 (5)	0.74526 (8)	0.0246 (2)	
O1	0.41858 (10)	0.24422 (5)	0.89266 (8)	0.0357 (2)	
C1	0.41307 (11)	0.21416 (6)	0.78858 (10)	0.0248 (2)	
C2	0.51580 (11)	0.24121 (6)	0.71036 (10)	0.0247 (2)	
H2	0.4683	0.2412	0.6211	0.030*	
C3	0.63773 (12)	0.18184 (7)	0.73050 (12)	0.0316 (3)	
H3A	0.6814	0.1789	0.8195	0.038*	
H3B	0.6021	0.1296	0.7032	0.038*	
C4	0.74652 (13)	0.20629 (8)	0.65716 (13)	0.0383 (3)	
H4A	0.8240	0.1693	0.6747	0.046*	
H4B	0.7053	0.2044	0.5675	0.046*	
C5	0.80004 (13)	0.28975 (8)	0.69353 (13)	0.0395 (3)	
H5A	0.8490	0.2905	0.7814	0.047*	
H5B	0.8654	0.3052	0.6430	0.047*	
C6	0.67966 (14)	0.34838 (7)	0.67307 (12)	0.0371 (3)	
H6A	0.6355	0.3506	0.5841	0.045*	
H6B	0.7154	0.4008	0.6992	0.045*	
C7	0.57121 (13)	0.32470 (7)	0.74771 (11)	0.0307 (3)	
H7A	0.4945	0.3622	0.7312	0.037*	
H7B	0.6134	0.3261	0.8372	0.037*	
C8	0.29903 (11)	0.12078 (6)	0.61624 (10)	0.0233 (2)	
H8	0.3593	0.1500	0.5715	0.028*	
C9	0.33861 (12)	0.03328 (7)	0.61448 (11)	0.0292 (3)	
H9A	0.2805	0.0024	0.6578	0.035*	
H9B	0.4352	0.0263	0.6586	0.035*	
C10	0.31939 (13)	0.00374 (7)	0.47898 (12)	0.0340 (3)	
H10A	0.3821	0.0323	0.4374	0.041*	
H10B	0.3430	−0.0521	0.4795	0.041*	
C11	0.16953 (14)	0.01560 (8)	0.40628 (12)	0.0369 (3)	
H11A	0.1076	−0.0167	0.4434	0.044*	
H11B	0.1606	−0.0014	0.3197	0.044*	
C12	0.12716 (15)	0.10224 (8)	0.40858 (12)	0.0381 (3)	
H12A	0.0298	0.1079	0.3662	0.046*	
H12B	0.1825	0.1337	0.3632	0.046*	
C13	0.14821 (12)	0.13330 (7)	0.54369 (11)	0.0286 (3)	
H13A	0.1259	0.1893	0.5419	0.034*	
H13B	0.0853	0.1058	0.5864	0.034*	
C14	0.23915 (12)	0.11819 (6)	0.82848 (10)	0.0257 (3)	
H14	0.1855	0.0750	0.7804	0.031*	
C15	0.13228 (13)	0.17564 (7)	0.86046 (12)	0.0334 (3)	
H15A	0.0738	0.1963	0.7833	0.040*	
H15B	0.1803	0.2199	0.9082	0.040*	
C16	0.04124 (15)	0.13386 (9)	0.93748 (13)	0.0417 (3)	
H16A	−0.0231	0.1717	0.9604	0.050*	
H16B	−0.0132	0.0928	0.8866	0.050*	
C17	0.12985 (17)	0.09710 (9)	1.05623 (13)	0.0473 (4)	
H17A	0.0699	0.0692	1.1015	0.057*	
H17B	0.1780	0.1387	1.1106	0.057*	
C18	0.23659 (16)	0.03963 (9)	1.02435 (13)	0.0445 (3)	
H18A	0.1883	−0.0047	0.9769	0.053*	
H18B	0.2947	0.0190	1.1017	0.053*	
C19	0.32894 (13)	0.08035 (8)	0.94690 (12)	0.0342 (3)	
H19A	0.3852	0.1208	0.9976	0.041*	
H19B	0.3915	0.0417	0.9230	0.041*	

### Atomic displacement parameters (Å^2^)

**Table:**

	*U*^11^	*U*^22^	*U*^33^	*U*^12^	*U*^13^	*U*^23^
N1	0.0262 (5)	0.0280 (5)	0.0215 (5)	−0.0046 (4)	0.0095 (4)	−0.0030 (3)
O1	0.0446 (5)	0.0404 (5)	0.0246 (4)	−0.0135 (4)	0.0129 (4)	−0.0098 (3)
C1	0.0266 (5)	0.0262 (5)	0.0217 (5)	−0.0017 (4)	0.0054 (4)	−0.0009 (4)
C2	0.0260 (5)	0.0269 (5)	0.0213 (5)	−0.0051 (4)	0.0053 (4)	−0.0025 (4)
C3	0.0292 (6)	0.0282 (6)	0.0379 (6)	−0.0031 (5)	0.0087 (5)	−0.0028 (5)
C4	0.0292 (6)	0.0391 (7)	0.0497 (8)	−0.0049 (5)	0.0151 (5)	−0.0089 (6)
C5	0.0312 (6)	0.0454 (7)	0.0439 (7)	−0.0142 (5)	0.0129 (5)	−0.0067 (6)
C6	0.0437 (7)	0.0309 (6)	0.0386 (7)	−0.0138 (5)	0.0133 (5)	−0.0035 (5)
C7	0.0360 (6)	0.0265 (6)	0.0309 (6)	−0.0062 (5)	0.0104 (5)	−0.0038 (4)
C8	0.0249 (5)	0.0251 (5)	0.0214 (5)	−0.0045 (4)	0.0083 (4)	−0.0027 (4)
C9	0.0288 (6)	0.0295 (6)	0.0291 (6)	0.0023 (4)	0.0059 (4)	−0.0039 (4)
C10	0.0374 (7)	0.0314 (6)	0.0344 (7)	−0.0002 (5)	0.0109 (5)	−0.0101 (5)
C11	0.0402 (7)	0.0367 (7)	0.0319 (6)	−0.0079 (5)	0.0039 (5)	−0.0111 (5)
C12	0.0425 (7)	0.0395 (7)	0.0276 (6)	0.0021 (5)	−0.0022 (5)	−0.0024 (5)
C13	0.0301 (6)	0.0276 (6)	0.0273 (6)	0.0012 (4)	0.0050 (5)	−0.0001 (4)
C14	0.0279 (6)	0.0278 (5)	0.0233 (5)	−0.0041 (4)	0.0101 (4)	−0.0010 (4)
C15	0.0339 (6)	0.0362 (6)	0.0345 (6)	0.0019 (5)	0.0171 (5)	0.0011 (5)
C16	0.0394 (7)	0.0505 (8)	0.0424 (7)	−0.0029 (6)	0.0245 (6)	−0.0021 (6)
C17	0.0567 (9)	0.0587 (9)	0.0332 (7)	−0.0126 (7)	0.0242 (6)	0.0014 (6)
C18	0.0524 (8)	0.0470 (8)	0.0359 (7)	−0.0061 (6)	0.0132 (6)	0.0137 (6)
C19	0.0342 (6)	0.0383 (7)	0.0307 (6)	−0.0018 (5)	0.0085 (5)	0.0064 (5)

### Geometric parameters (Å, º)

**Table:**

N1—C1	1.3634 (14)	C10—C11	1.5212 (18)
N1—C8	1.4785 (13)	C10—H10A	0.9700
N1—C14	1.4826 (13)	C10—H10B	0.9700
O1—C1	1.2317 (13)	C11—C12	1.5218 (18)
C1—C2	1.5283 (15)	C11—H11A	0.9700
C2—C7	1.5322 (14)	C11—H11B	0.9700
C2—C3	1.5392 (16)	C12—C13	1.5316 (16)
C2—H2	0.9800	C12—H12A	0.9700
C3—C4	1.5267 (17)	C12—H12B	0.9700
C3—H3A	0.9700	C13—H13A	0.9700
C3—H3B	0.9700	C13—H13B	0.9700
C4—C5	1.5243 (18)	C14—C15	1.5252 (16)
C4—H4A	0.9700	C14—C19	1.5300 (16)
C4—H4B	0.9700	C14—H14	0.9800
C5—C6	1.520 (2)	C15—C16	1.5279 (16)
C5—H5A	0.9700	C15—H15A	0.9700
C5—H5B	0.9700	C15—H15B	0.9700
C6—C7	1.5302 (17)	C16—C17	1.521 (2)
C6—H6A	0.9700	C16—H16A	0.9700
C6—H6B	0.9700	C16—H16B	0.9700
C7—H7A	0.9700	C17—C18	1.524 (2)
C7—H7B	0.9700	C17—H17A	0.9700
C8—C9	1.5279 (15)	C17—H17B	0.9700
C8—C13	1.5302 (15)	C18—C19	1.5323 (17)
C8—H8	0.9800	C18—H18A	0.9700
C9—C10	1.5286 (16)	C18—H18B	0.9700
C9—H9A	0.9700	C19—H19A	0.9700
C9—H9B	0.9700	C19—H19B	0.9700
			
C1—N1—C8	124.39 (9)	C11—C10—H10B	109.5
C1—N1—C14	119.73 (9)	C9—C10—H10B	109.5
C8—N1—C14	115.84 (8)	H10A—C10—H10B	108.1
O1—C1—N1	121.32 (10)	C10—C11—C12	110.75 (10)
O1—C1—C2	119.40 (10)	C10—C11—H11A	109.5
N1—C1—C2	119.13 (9)	C12—C11—H11A	109.5
C1—C2—C7	111.49 (9)	C10—C11—H11B	109.5
C1—C2—C3	108.41 (9)	C12—C11—H11B	109.5
C7—C2—C3	109.97 (9)	H11A—C11—H11B	108.1
C1—C2—H2	109.0	C11—C12—C13	111.45 (10)
C7—C2—H2	109.0	C11—C12—H12A	109.3
C3—C2—H2	109.0	C13—C12—H12A	109.3
C4—C3—C2	111.28 (10)	C11—C12—H12B	109.3
C4—C3—H3A	109.4	C13—C12—H12B	109.3
C2—C3—H3A	109.4	H12A—C12—H12B	108.0
C4—C3—H3B	109.4	C8—C13—C12	110.87 (10)
C2—C3—H3B	109.4	C8—C13—H13A	109.5
H3A—C3—H3B	108.0	C12—C13—H13A	109.5
C5—C4—C3	110.79 (10)	C8—C13—H13B	109.5
C5—C4—H4A	109.5	C12—C13—H13B	109.5
C3—C4—H4A	109.5	H13A—C13—H13B	108.1
C5—C4—H4B	109.5	N1—C14—C15	113.01 (9)
C3—C4—H4B	109.5	N1—C14—C19	112.78 (9)
H4A—C4—H4B	108.1	C15—C14—C19	111.69 (10)
C6—C5—C4	110.57 (10)	N1—C14—H14	106.2
C6—C5—H5A	109.5	C15—C14—H14	106.2
C4—C5—H5A	109.5	C19—C14—H14	106.2
C6—C5—H5B	109.5	C14—C15—C16	110.43 (10)
C4—C5—H5B	109.5	C14—C15—H15A	109.6
H5A—C5—H5B	108.1	C16—C15—H15A	109.6
C5—C6—C7	111.31 (10)	C14—C15—H15B	109.6
C5—C6—H6A	109.4	C16—C15—H15B	109.6
C7—C6—H6A	109.4	H15A—C15—H15B	108.1
C5—C6—H6B	109.4	C17—C16—C15	111.15 (11)
C7—C6—H6B	109.4	C17—C16—H16A	109.4
H6A—C6—H6B	108.0	C15—C16—H16A	109.4
C6—C7—C2	110.33 (10)	C17—C16—H16B	109.4
C6—C7—H7A	109.6	C15—C16—H16B	109.4
C2—C7—H7A	109.6	H16A—C16—H16B	108.0
C6—C7—H7B	109.6	C16—C17—C18	111.00 (11)
C2—C7—H7B	109.6	C16—C17—H17A	109.4
H7A—C7—H7B	108.1	C18—C17—H17A	109.4
N1—C8—C9	112.66 (9)	C16—C17—H17B	109.4
N1—C8—C13	111.86 (9)	C18—C17—H17B	109.4
C9—C8—C13	110.29 (9)	H17A—C17—H17B	108.0
N1—C8—H8	107.2	C17—C18—C19	111.22 (11)
C9—C8—H8	107.2	C17—C18—H18A	109.4
C13—C8—H8	107.2	C19—C18—H18A	109.4
C8—C9—C10	110.50 (9)	C17—C18—H18B	109.4
C8—C9—H9A	109.6	C19—C18—H18B	109.4
C10—C9—H9A	109.6	H18A—C18—H18B	108.0
C8—C9—H9B	109.6	C14—C19—C18	110.50 (10)
C10—C9—H9B	109.6	C14—C19—H19A	109.6
H9A—C9—H9B	108.1	C18—C19—H19A	109.6
C11—C10—C9	110.87 (10)	C14—C19—H19B	109.6
C11—C10—H10A	109.5	C18—C19—H19B	109.6
C9—C10—H10A	109.5	H19A—C19—H19B	108.1
			
C8—N1—C1—O1	172.53 (10)	N1—C8—C9—C10	−176.70 (9)
C14—N1—C1—O1	−5.19 (16)	C13—C8—C9—C10	57.54 (12)
C8—N1—C1—C2	−11.91 (16)	C8—C9—C10—C11	−57.93 (13)
C14—N1—C1—C2	170.37 (9)	C9—C10—C11—C12	56.68 (14)
O1—C1—C2—C7	−23.63 (15)	C10—C11—C12—C13	−55.58 (15)
N1—C1—C2—C7	160.73 (10)	N1—C8—C13—C12	177.46 (9)
O1—C1—C2—C3	97.56 (12)	C9—C8—C13—C12	−56.32 (12)
N1—C1—C2—C3	−78.08 (12)	C11—C12—C13—C8	55.60 (14)
C1—C2—C3—C4	−178.71 (9)	C1—N1—C14—C15	65.94 (13)
C7—C2—C3—C4	−56.59 (12)	C8—N1—C14—C15	−111.97 (11)
C2—C3—C4—C5	56.50 (14)	C1—N1—C14—C19	−61.92 (13)
C3—C4—C5—C6	−56.37 (15)	C8—N1—C14—C19	120.17 (10)
C4—C5—C6—C7	57.27 (14)	N1—C14—C15—C16	175.50 (10)
C5—C6—C7—C2	−57.68 (13)	C19—C14—C15—C16	−56.07 (14)
C1—C2—C7—C6	176.91 (9)	C14—C15—C16—C17	56.40 (15)
C3—C2—C7—C6	56.63 (12)	C15—C16—C17—C18	−56.75 (16)
C1—N1—C8—C9	118.68 (11)	C16—C17—C18—C19	56.23 (16)
C14—N1—C8—C9	−63.52 (12)	N1—C14—C19—C18	−175.89 (10)
C1—N1—C8—C13	−116.41 (11)	C15—C14—C19—C18	55.56 (14)
C14—N1—C8—C13	61.39 (12)	C17—C18—C19—C14	−55.32 (15)

### Hydrogen-bond geometry (Å, º)

**Table:**

*D*—H···*A*	*D*—H	H···*A*	*D*···*A*	*D*—H···*A*
C2—H2···O1^i^	0.98	2.44	3.3861 (13)	163

Symmetry code: (i) *x*, −*y*+1/2, *z*−1/2.

## References

[bb1] Agilent (2012). *CrysAlis PRO* and *CrysAlis RED* Agilent Technologies, Yarnton, England.

[bb2] Cremer, D. & Pople, J. A. (1975). *J. Am. Chem. Soc.* **97**, 1354–1358.

[bb3] Saeed, S., Jasinski, J. P. & Butcher, R. J. (2011*a*). *Acta Cryst.* E**67**, o279. 10.1107/S1600536811000122 PMC3051426 21522971

[bb4] Saeed, S., Rashid, N., Hussain, R. & Wong, W.-T. (2012). *Acta Cryst.* E**68**, o26. 10.1107/S1600536811051464 PMC3254366 22259531

[bb5] Saeed, S., Rashid, N., Ng, S. W. & Tiekink, E. R. T. (2011*b*). *Acta Cryst.* E**67**, o1194. 10.1107/S1600536811014450 PMC3089125 21754496

[bb6] Sheldrick, G. M. (2008). *Acta Cryst.* A**64**, 112–122. 10.1107/S0108767307043930 18156677

